# Training Set Augmentation and Harmonization Enables Radiomic Models to Detect Early Onset of Lung Cancer

**DOI:** 10.21203/rs.3.rs-7350820/v1

**Published:** 2025-09-29

**Authors:** Claire Huchthausen, Menglin Shi, Gabriel L.A. Sousa, James Larner, Einsley Janowski, Jonathan Colen, Krishni Wijesooriya

**Affiliations:** University of Virginia; University of Virginia; University of Virginia; University of Virginia; University of Virginia; Old Dominion University; University of Virginia

**Keywords:** lung cancer, diagnosis, radiomics, harmonization, data augmentation, machine learning

## Abstract

Radiomics-based machine learning models have the potential to detect lung cancer at inception from CT scans and transform patient outcomes. Low malignancy rates in early-development pulmonary nodules (PNs) and variable image acquisition hinder development of clinically applicable radiomics-based early detection models. To address these challenges, we augmented training using later-development PNs and harmonized for acquisition effects. We first trained machine learning models to predict PN malignancy using radiomic features from scans of early-development benign and malignant PNs (n = 187) harmonized using ComBat. Observing near-chance performance, we augmented training with later-development benign and malignant PNs (n = 225). We evaluated whether harmonization must incorporate biological differences that impact acquisition effects in added training data. To correct features for variability in four acquisition parameters, we compared: 1) harmonization without biological distinction, 2) harmonizing with a covariate distinguishing early-development, benign augmentation, malignant augmentation training datasets, 3) harmonizing each dataset separately. Models trained using augmented data harmonized without biological distinction failed to improve. Models trained on augmented data harmonized with a covariate (ROC-AUC 0.72 [0.67–0.76]) or separately (ROC-AUC 0.69 [0.63–0.74]) achieved significantly higher test ROC-AUC (Delong test, adjusted p ≤ 0.05). Our findings lay groundwork for clinically viable radiomics tools harnessing routine screening imaging for lung cancer early detection.

## Introduction

Lung cancer causes one in five cancer deaths worldwide [1]. Patients whose lung cancer is detected at an early stage when the cancer is localized exhibit a 61% five-year relative survival, compared to 7% in those whose cancer is undetected until it has distant metastasis [2]. Even before metastasis, larger tumors lead to higher treatment-related toxicity [3]. Adults at risk for lung cancer undergo annual screening (LCS) with computed tomography (CT) scans, where physicians identify abnormal growths or pulmonary nodules (PNs) and mark these as suspicious for cancer if they meet certain criteria [4]. But common indicators such as size and rate of growth are sometimes caused by benign inflammatory processes [5], and visual cues such as smoothness, irregularity, and speculation are subject to each physician’s interpretation [6]. A definitive diagnosis requires follow-up scans over several months, plus Positron Emission Tomography (PET) scans or biopsy.

Medical images contain information indiscernible to the naked eye [6],[7],[8] which can be extracted using radiomic features, a set of statistical and spatial interrelationships between selected voxels in an image [7]. Machine learning (ML) models can harness these radiomic features to predict clinical outcomes [6],[8]. Radiomics-based ML could potentially detect lung cancer earlier than the current standard of care. However, there are two major barriers to creating radiomics-based early detection models in a clinical context. First, radiomic features are sensitive to image acquisition protocols [9] which vary across and within clinics. These non-biological sources of variation make it difficult to meaningfully combine data or reproduce results [10]. Second, there is a low occurrence rate of cancer in PNs which are very small or early in their development—the target PNs in the early detection problem. For example, when Lung-RADS classifications were applied to scans from the National Lung Screening Trial in a retrospective study, only 0.39% of patients with PNs classified as benign or probably benign at baseline would be diagnosed with cancer after follow-up [11].

To address this class imbalance and potentially improve model performance, ML training data may be augmented with radiomic features from confirmed benign and malignant PNs later in their development. However, including these PNs in a training set may exacerbate the challenge of radiomic features’ dependence on acquisition protocols, especially when biological differences in later-development benign and malignant PNs cause their scans to be affected differently by acquisition protocols. For example, contrast enhancement (CE), administered during scans for cases with clinical concern [12], tends to accumulate more in tumors than in healthy tissue [13]. Both the frequency and the effect of CE create a quasi-systematic difference between later-development benign and malignant PNs that their radiomic features might inherit when calculated from routine imaging.

To mitigate effects of variable image acquisition parameters and improve generalizability of models, ComBat (“Combine Batches”) harmonization can learn corrective transformations to remove acquisition-related bias [14],[15] from radiomic feature distributions [16],[17],[18]. In CT-based radiomics for lung cancer, ComBat has been applied mostly in predictive pipelines addressing outcomes like recurrence [19], development-free survival [20], and overall survival [21]. These predictive problems use data from only malignant PNs. In contrast, lung cancer diagnosis models require data from both benign and malignant PNs. Some studies have implemented ComBat when combining radiomic data from benign and malignant tissues, but most of these have corrected for multi-center overall effects rather than variation in specific acquisition protocols [22], [23], [24]. The challenge of harmonizing radiomic features from benign and malignant tissues resembles the challenge of harmonizing features from tissues of different origin. Previous studies have considered methods of applying ComBat to harmonize radiomic feature data from tissues of different origin (breast tumor and healthy liver tissue) when acquisition protocols vary, including harmonizing with tissue type as a covariate [25] and harmonizing different tissue types separately [25],[26]. The best practice for harmonizing benign and malignant forms of the same tissue—such as lung tumor and benign lung tissue—for specific acquisition protocols has not yet been established.

To develop radiomics-based models that can accurately predict the malignancy of PNs at their earliest development from routine clinical imaging, we augmented training with later-development benign and malignant PNs and accounted for non-standardized acquisition using ComBat harmonization. To remove radiomic features’ dependence on four acquisition parameters, we compared three methods of harmonizing augmented training sets: 1) harmonizing all training data collectively in the standard manner, with no biological distinction, 2) harmonizing with a covariate to distinguish early-development data, later-development benign augmentation data, and later-development malignant augmentation data, and 3) harmonizing these three datasets separately. We compared the performance of lung cancer early detection models trained on augmented data harmonized using these methods.

## Results

### Baseline radiomic model for early detection

To evaluate radiomics-based early detection of lung cancer, we collected a dataset containing clinical chest CT scans of early-development PNs. Because physicians primarily interpret scans based on PN size and rate of growth, we use physicians’ interpretation at the time of a given scan as an indicator of PN development. For the purposes of this study, all PNs interpreted at time of scan as benign, probably benign, or indeterminate are considered early-development. Each PN was scanned multiple times separated by interims of months, and each scan had a corresponding time-of-scan interpretation.

The PNs imaged in the early-development scans received follow-up. At the end of follow-up, some PNs were diagnosed as benign and some PNs were diagnosed as malignant (details in Methods). We refer to this as a PN’s endpoint diagnosis, which may differ from its time-of-scan diagnoses (for examples, see Supplementary Fig. S1). The early-development scans of PNs with an endpoint malignant diagnosis show the earliest onset of lung cancer. Overall, the early-development dataset had only 16.6% incidence of cancer (Table 1). Throughout this study, we evaluate model performance using held-out test data drawn only from this early-development dataset.

**Figure 1:**
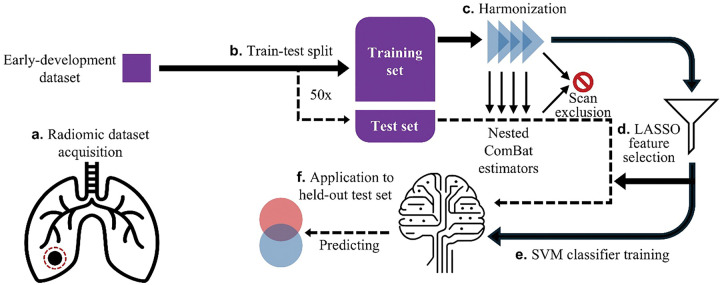
The pipeline for our baseline early detection model. The solid line represents the training set and the dashed line represents the test set for a given trial. An arrow pointing from the training set to the test set indicates that a corresponding result from training set processing is used to process the test set.

We developed a predictive pipeline using this data, shown schematically in Fig. 1. PNs were segmented in the CT images using semi-automated contouring software. Scans were preprocessed (see Methods) and a total of 107 radiomic features were extracted for each scan of a PN using the open-source Python library PyRadiomics (Fig. 1a) [8]. This early-development dataset was randomly split into five folds while ensuring no patients overlap between training and test sets, repeated 10 times (50 trials; Fig. 1b).

The dataset included scans acquired using non-standardized parameters, including kilovoltage peak (KVP), focal spots, scanner manufacturer, and CE. For each training set, we used Optimized Permutation Nested ComBat (OPNCB), which applies ComBat to remove biases from multiple protocols sequentially [27], to correct for variability in these four parameters (Fig. 1c). Scans with rare acquisition protocols were excluded from a trial when ComBat could not learn a correction due to insufficient samples of that protocol.

To predict PN endpoint diagnosis from harmonized radiomic features, we trained a two-stage predictive model. First, a least absolute shrinkage and selection operator (LASSO) selected a subset of predictive features (Fig. 1d) [28]. From all radiomic features, LASSO selected an average of 3.8 [3.3–4.4] (n = 49; brackets indicate the 95% confidence interval here and henceforth) features per trial. Next, a linear Support Vector Machine (SVM) [28] used selected features from the training set to classify PNs by endpoint diagnosis, benign or malignant (Fig. 1e).

The trained ComBat estimators, feature selection results, and trained SVM were applied to the held-out test set for each trial (Fig. 1F). The ComBat algorithm failed for one trial’s test set due to insufficient sample sizes within the test set, so this trial was excluded from all further consideration in this study. This model trained on only early-development data achieved an average ROC-AUC of 0.58 [0.51–0.64] (n = 49) for the test set. This performance is near chance, motivating our hypothesis that augmenting the training set with additional data may improve early detection model performance.

### Training set augmentation with radiomic features from later-development PNs

**Figure 2:**
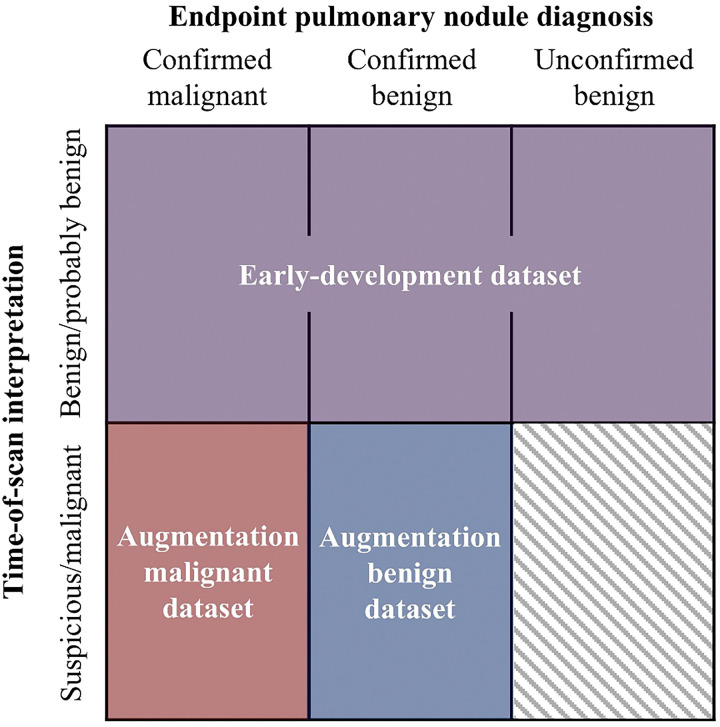
Definitions of how scans were classified into datasets. Each cell represents a category of scans defined by the intersection of the axes labels, with colors indicating the dataset to which that category was assigned. The greyed-out region indicates a category which does not exist because all PNs interpreted as suspicious/malignant in our datasets received follow-up to confirm diagnosis.

**Figure 3:**
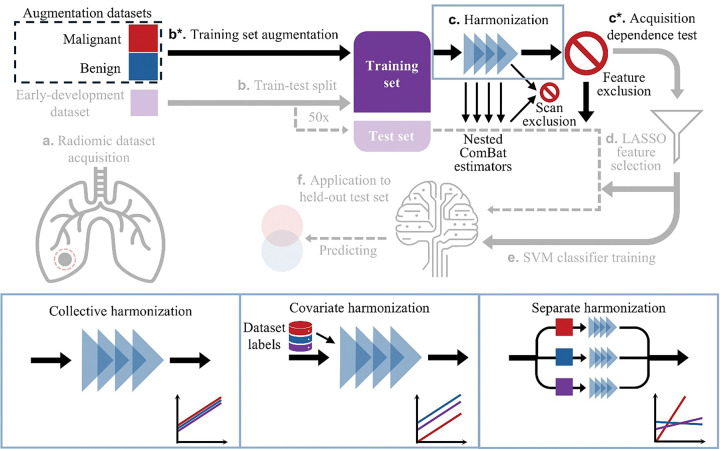
The pipeline for our early detection models with augmented training sets. Pipeline elements with reduced opacity are procedures that remain unchanged from Fig. 1. Asterisks (*) indicate new steps in the pipeline as compared to Fig. 1. The solid line represents the training set and the dashed line represents the test set for a given trial. An arrow pointing from the training set to the test set indicates that a corresponding result from training set processing is used to process the test set. Figure 3c represents one of the three harmonization methods expanded schematically at the bottom of the figure. Within the expansions (bottom), line graphs illustrate the type of corrective transformations in feature space that each method calculates.

To improve performance, we added radiomic feature data from benign and malignant PNs later in their development (n = 225 scans, see Table 1) to the early-development training data. For the purposes of this study, PNs interpreted at time of scan as suspicious or malignant are later-development. We refer to later-development benign and malignant scans as augmentation datasets because they augment early-development training data but are not used to test performance. We will refer to training sets that contain data from both the early-development dataset and the augmentation datasets as augmented training sets. Figure 2 visualizes our scan classification scheme, where the early-development dataset is shown in purple. Supplementary Figure S1 follows three representative PNs to further clarify our classification scheme. The malignant dataset holds later-development scans of PNs that were later confirmed as malignant using PET/CT or biopsy (red in Fig. 2). The benign dataset holds later-development scans of PNs that were later confirmed as benign using wedge resection or biopsy (blue in Fig. 2). Both augmentation datasets include follow-up scans of PNs that have previous scans in the early-development dataset. If a patient appeared in a trial’s test set, no later-development scans from that patient were included in that trial’s training set. When augmentation datasets were included, occurrence of cancer increased from 16.6–53.4% in our total data pool (Table 1), removing the previous class imbalance. We added these augmentation datasets to the original early-development training sets in all trials and trained early detection models following the pipeline in Fig. 3.

**Figure 4:**
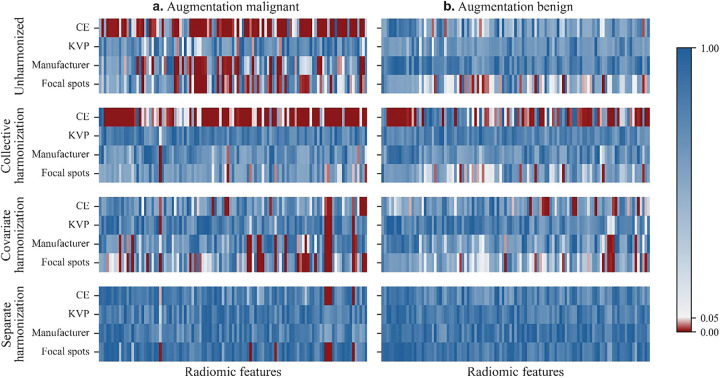
Acquisition dependence of radiomic features defined by the Kruskal-Wallis test in an example training set. *P*-values ≤0.05, indicating dependence of a feature (107 total, x-axes) on an acquisition parameter (y-axes), are in red. More red cells in a row show a greater frequency of dependence on the corresponding acquisition parameter. a. Acquisition dependence within scans from the augmentation malignant dataset. b. Acquisition dependence within scans from the augmentation benign dataset.

Both augmentation datasets were also highly variable in our acquisition parameters of interest (Supplementary Table S1). To evaluate acquisition dependence of radiomic features in each augmented training set, we developed a procedure using the Kruskal-Wallis test [17], see Methods. A significant result (p ≤ 0.05) indicated that a given feature was acquisition-dependent. Table 2 summarizes feature dependence of acquisition parameters and Fig. 4 shows *p*-values for acquisition dependence of each feature and acquisition parameter from a sample trial. Without harmonization, 12.1% [11.2–13.1%] of radiomic features were acquisition-independent (n = 49 trials, Fig. 4 top row). To mitigate effects of acquisition variation within each trial, we applied OPNCB to learn corrective transformations from the entire augmented training set (hereafter: collective harmonization, Fig. 3 bottom left), and features which remained acquisition dependent were excluded from further analysis in each trial (Fig. 3c*). However, collective harmonization lowered the proportion of acquisition-independent features to 2.6% [2.2–3.1%] (n = 49 trials; Wilcoxon signed-rank, *p* = 1.6e-08). Collective harmonization increased CE dependence, and consequently total acquisition dependence, compared to unharmonized data for both benign and malignant augmentation datasets (Table 2 and Fig. 4 second row).

### Comparing harmonization methods to remove acquisition dependence in augmented training sets

Collective harmonization assumed each acquisition protocol biased a radiomic feature in the same way across all three datasets, yet this assumption may be invalid due to biological and acquisitional differences between early-development, later-development benign, and later-development malignant datasets. Radiomic features in later-development benign and malignant datasets may have different distributions due to their biological differences independent of acquisition. Specific protocols are used to acquire scans in different datasets at different frequencies (Supplementary Table S1), further influencing the feature distributions. Using ComBat with a covariate—an added corrective parameter learned for a specific dataset—accounts for this difference in distributions, while still assuming a protocol has the same effect on scans in different datasets [25]. A transformation applied to scans in different datasets has the same slope but a net shift in intercept [25].

When a protocol has different effects on scans in different datasets, corresponding corrective transformations may have to differ between datasets in both intercept and slope. Because ComBat estimates acquisition effects from all input data, datasets with different acquisition effects (additive or multiplicative) should be harmonized separately [25]. For example, different tissue types (breast tumor and healthy liver tissue) require distinct ComBat transformations to correct for acquisition variability [25] due to their biological differences.

CE exemplifies a difference in both the means and acquisition effects between our later-development benign and malignant augmentation datasets. Later-development scans administered with CE due to clinical concern are more likely to be malignant than benign (Supplementary Table S1), creating a difference in mean for some radiomic features, and contrast medium is expected to accumulate more in tumors than in healthy tissues [25], creating a difference in acquisition effect for some radiomic features. Collectively harmonized features exhibited frequent acquisition dependence due to CE, indicating that collective harmonization does not handle these differences well.

Thus, to account for baseline and acquisition differences between biologically distinct PNs, we compared the additional methods of applying the OPNCB algorithm: 1) harmonizing with a covariate to distinguish early-development, late-development benign, and late-development malignant datasets (hereafter: covariate harmonization, Fig. 3 bottom center), or 2) harmonizing early-development, late-development benign, and late-development malignant datasets separately (hereafter: separate harmonization, Fig. 3 bottom right).

When all augmentation data was added to the training set (Fig. 3c*), the proportion of acquisition-independent features defined by Kruskal-Wallis averaged 12.3% [11.4–13.3%] (n = 49 trials) with covariate harmonization, and 86.9% [84.4–89.5%] (n = 49 trials) with separate harmonization. The frequency of each radiomic feature’s net acquisition dependence after each method—how often the distribution of that feature was dependent on at least one acquisition parameter—is given in Supplementary Table S2. When comparing metrics for the three methods, adjusted *p*-values were calculated using Holm-Bonferroni. Covariate harmonization significantly increased the proportion of acquisition-independent features compared to collective harmonization (Wilcoxon signed-rank, adjusted *p* = 1.4e-09, *n* = 49). Separate harmonization significantly increased the proportion of acquisition-independent features compared to both collective harmonization (Wilcoxon signed-rank, adjusted *p* = 1.4e-09, *n =* 49) and covariate harmonization (Wilcoxon signed-rank, adjusted *p* = 1.9e-115, *n =* 49).

### Impact of training set augmentation and harmonization method on early detection models

To observe how the performance of early detection models scales with the amount of added training data, we constructed a learning curve by varying the fraction of the augmentation datasets added to each training set. For each level of augmentation of each training set, LASSO-SVM models were trained on features harmonized using collective, covariate, or separate harmonization, creating three versions of a model for each augmented training set. We refer to these models as collective, covariate, and separate harmonization models depending on the harmonization method applied during training.

While covariate and separate harmonization distinguish benign and malignant PNs in the augmentation datasets, they make no such distinction when harmonizing the early-development dataset. Applying the trained ComBat estimators to the test set, which is drawn only from the early-development dataset, uses no foreknowledge of whether each PN is benign or malignant. Thus, test set performance simulates clinical application for early detection where the endpoint diagnosis is unknown.

**Figure 5:**
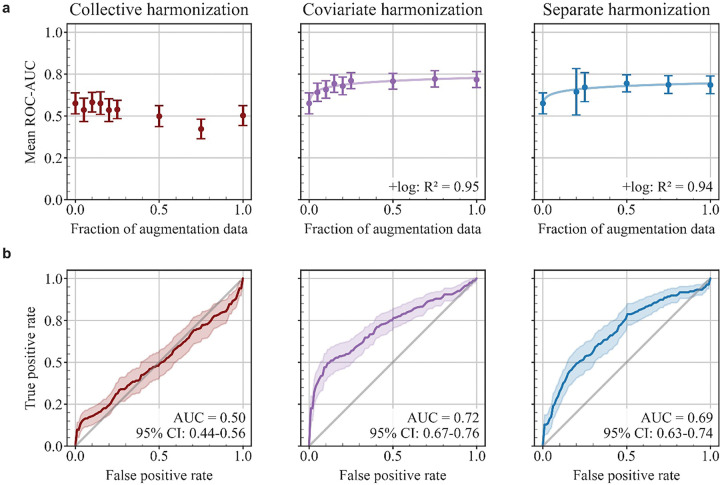
The average testing performance of LASSO-SVM early detection models. a. The learning curves for models with augmented training sets obtained by varying the fraction of augmentation data included in the training set. The leftmost point in each plot represents the baseline un-augmented model. Error bars show the 95% confidence interval over trials where a model could be trained. Trial numbers *n* for each point, from left to right—collective harmonization: *n*=[49, 49, 49, 49, 48, 49, 47, 42, 45], covariate harmonization: *n*=[49, 49, 49, 49, 49, 49, 49, 49, 49], separate harmonization: *n*=[49, 13, 29, 49, 49, 49]. Separate harmonization failed for 5%, 10%, and 15% augmentation due to small sample size, so these points are omitted. b. Receiver operating curves for models trained using 100% of augmentation data, averaged over all trials where a model could be trained. Shaded regions show the 95% confidence interval. Trial numbers for each curve—collective harmonization: *n*=45, covariate harmonization: *n*=49, separate harmonization: *n*=49.

When increasing the amount of augmentation data included in training, we found that collective harmonization models’ performance failed to improve (Fig. 5a left). In contrast, accounting for biological differences when harmonizing augmentation data enabled LASSO-SVM models to improve as more training data was added. We observed logarithmic scaling between augmentation fraction and test ROC-AUC for both covariate ( *R*^2^ = 0.95, Fig. 5a center) and separate ( *R*^2^ = 0.94, Fig. 5a right) harmonization models.

When 100% of augmentation data were added to the training set, models achieved average test ROC-AUC of 0.50 [0.44–0.56] (n = 45) for collective harmonization, 0.72 [0.67–0.76] (n = 49) for covariate harmonization, and 0.69 [0.63–0.74] (n = 49) for separate harmonization (Fig. 5B). All but one of collective harmonization models could not separate benign and malignant classes and classified all samples as benign. According to the Delong test, the difference between test ROC-AUC of separate and collective harmonization models was significant (adjusted *p* ≤ 0.05) for 24.4% of trials (n = 45), where separate harmonization outperformed collective harmonization. The difference between covariate and collective harmonization models was also significant for 28.8% of trials (n = 45), where covariate harmonization outperformed collective harmonization. The difference between separate and covariate harmonization models was significant for 6.1% of trials (n = 49), where covariate harmonization outperformed separate harmonization. The weighted accuracy, sensitivity, and specificity of predictions on test samples are reported in Table 3. Covariate harmonization models outperformed separate harmonization in sensitivity (Wilcoxon signed-rank, *p =* 0.014, n = 49) but covariate and separate harmonization models did not show a significant difference in specificity.

### Predictive features in early detection models with augmented training sets

We again focus our analysis on trials where 100% of augmentation data were added to the training set. From features which were acquisition-independent post-harmonization, LASSO selected an average of 1.0 [0.8–1.3] (n = 45) features per trial for collective harmonization, 3.5 [3.0–4.1] (n = 49) for covariate harmonization, and 7.4 [6.7–8.3] (n = 49) for separate harmonization. The most frequently selected features varied by harmonization method: Flatness for collective harmonization, GLCM informational measure of correlation 1 for covariate harmonization, and Flatness, Sphericity, NGTDM Strength, and GLDM Small Dependence High Gray Level Emphasis for separate harmonization. Some of these features have been related to lung cancer [29], [30]. Complete feature selection frequencies are detailed in Supplementary Table S3.

## Discussion

The value of a radiomics-based machine learning model for lung cancer early detection is determined by its applicability to clinical scenarios, which must overcome both an inherent sample class imbalance and non-standardized image acquisition. In this study, we addressed class imbalance by augmenting training data with later-development benign and malignant PNs and addressed acquisition variability using ComBat harmonization. Correcting acquisition effects is essential for clinical utility because models trained on acquisition-dependent radiomic features do not generalize well to new sites [31],[25] or rare acquisition protocols. Models trained without harmonization—using acquisition-dependent data—may appear to exhibit good performance. Using our datasets, LASSO-SVMs with unharmonized augmented training sets achieved an average ROC-AUC of 0.76 [0.71,0.81] (n = 49) on unharmonized test sets. But this outcome is likely an artifact of site-specific acquisition effects that differ distinctively between benign and malignant PNs. These effects prevent such a model from being generalizable and are precisely what harmonization aims to remove.

Harmonization becomes complicated when augmenting training data with later-development PNs because radiomic features from biologically distinct PNs—early-development PNs, later-development benign PNs, later-development malignant PNs—may each need distinct corrections to compensate for acquisition variability. Returning to our previous example, CE may have a large effect on feature distributions from later-development malignant PNs, which have enhanced permeability and retention, while having a smaller effect on features from later-development benign PNs (e.g. see graph for unharmonized data in Fig. 4). While we focus our analysis on differences across later-development datasets, features of early-development PNs may also show distributions distinct from those of later-development PNs, one example being PN volume.

Incorrectly harmonized radiomic features may mislead a model rather than improve performance. Indeed, when harmonization did not distinguish biologically distinct later-development PNs, we found that performance decreased with larger amounts of augmentation data. Thus, we evaluated methods of distinguishing biologically distinct PNs during harmonization. While collective harmonization applied identical corrections to all datasets, separate and covariate harmonization learned corrections specific to each biologically distinct dataset (Fig. 3). Covariate harmonization accounted for dataset-specific feature distributions and separate harmonization further accounted for dataset-specific acquisition effects. Both separate and covariate harmonization recovered acquisition-independent distributions in benign and malignant augmentation training data for significantly more features than collective harmonization, indicating that radiomic features of later-development benign and malignant PNs need distinct corrections. Separate harmonization removed acquisition dependence from significantly more features than covariate harmonization, indicating that acquisition protocols have different effects on benign and malignant PNs for many radiomic features (Supplementary Table S2).

Accordingly harmonized radiomic features from benign and malignant PNs could effectively augment the training of lung cancer early detection models. However, because separate harmonization partitions the data into smaller subsets before learning corrections, its corrections are limited by a smaller sample size than covariate harmonization [25]. If we increase sample size by relaxing our scan inclusion criteria and adding benign PNs that did not receive follow-up (n = 156) to the early-development dataset, the performance of 100% augmented separate harmonization models (ROC-AUC 0.72 [0.69–0.76]) matches that of 100% augmented covariate harmonization models (ROC-AUC 0.73 [0.68–0.77]). Overcoming these sample size limitations and confirming our results on a larger dataset is a promising direction of future study.

The impacts of our acquisition parameters of interest have been studied [9], [32], [33], [34]. Our datasets are heterogeneous in several additional acquisition parameters (e.g. scanner model and convolution kernel [33]) which could not be controlled for in this study due to high variation and consequently small sample size for specific protocols. Further, lung cancer type (e.g. squamous cell, adenocarcinoma) for malignant scans was not uniform and these biological differences may also impact acquisition effects within malignant PNs.

This study aimed to closely simulate a clinical application scenario for radiomics-based lung cancer early detection. Our datasets were gathered from a clinical setting and had high acquisition variability, providing a realistic test of harmonization methods for a clinical early detection scenario. While covariate and separate harmonization account for biological differences in augmentation training data, the endpoint diagnosis is unknown when harmonizing and classifying scans in the early-development dataset, as in the clinic. Scans in our early-development dataset are those actually seen in clinical early detection efforts. As such, some uncertainty in the endpoint diagnosis of these scans, and thus the target classification for the ML model, is inherited from the clinical process. This uncertainty is obvious for PNs when their benign endpoint diagnosis is unconfirmed. To mitigate this uncertainty, we include unconfirmed benign PNs only when at least two scans were performed to demonstrate stability over time, but this remains a limitation of our study. Nonetheless, such PNs constitute most of what is seen in lung cancer screening and including these in our dataset faithfully simulates the clinical scenario. Early-development scans with a malignant endpoint diagnosis also have an uncertain ML classification because the PN may not have developed malignant characteristics by the time of scan. On the other hand, these scans are central to modeling lung cancer detection at its earliest onset.

To meaningfully combine radiomic feature data from early-development and later-development PNs and harness this information for early lung cancer detection, we must first establish methods to remove acquisition dependence from these different data types. Based on our analysis, we recommend harmonizing radiomic features from early-development, later-development benign, and later-development malignant PNs either separately or with a covariate. Our finding that acquisition effects differ between benign and malignant tissues of the same tissue origin may extend to other cancer types and investigating this is a direction of future study. Studying these harmonization methods is an important step towards radiomics as a clinically viable quantitative tool for detecting lung cancer at its earliest onset, which could allow malignant tumors to be treated months earlier and reduce unnecessary resection and imaging procedures, reducing healthcare costs and improving patient outcomes.

## Methods

### Radiomic dataset acquisition

This retrospective study was approved by the UVA Institutional Review Board (IRB) (approval number: 301447), and due to its retrospective nature, informed consent was waived by the UVA IRB. All methods were performed in accordance with the relevant guidelines and regulations. We acquired chest CT scans of PNs from the University of Virginia hospital. Physician annotations and comments on scans were available for all PNs. If resection, biopsy, or PET/CT were performed, these results were available. Some patients had multiple PNs and some PNs had multiple follow-up scans separated by interims of months (Table 1).

All PNs in the early-development dataset were interpreted as benign, probably benign, or indeterminate at time of scan. Keyword indicators of this physician interpretation were, in order of the priority with which they were used to classify scans: benign (Lung-RADS category 2), probably benign (Lung-RADS category 3), inflammatory/infectious, indeterminate, 12-month follow-up (without verbal indication of suspicion), 6-month follow-up (without verbal indication of suspicion).

After follow-up, PNs from scans in the early-development dataset either received a malignant diagnosis, confirmed by biopsy or PET/CT, a benign diagnosis, confirmed by biopsy or wedge resection, or showed no indicators of becoming malignant. These last PNs are presumed benign, supported by at least one follow-up scan but without an additional diagnosis method.

All scans in our augmentation datasets were interpreted as suspicious or malignant at time of scan. Keyword indicators of this physician interpretation were, in order of priority: malignant, known cancer, very suspicious (Lung-RADS category 4B), suspicious (Lung-RADS category 4A), possibility for neoplasm, recommendation for PET/CT or biopsy (without verbal indication of indeterminate or alternative diagnosis).

The malignant augmentation dataset holds scans of PNs that were later confirmed as malignant by biopsy or PET/CT. The benign augmentation dataset holds scans of PNs that were later confirmed as benign by biopsy or wedge resection.

CT images for all datasets were acquired in DICOM format. CE information was noted from scan protocol comments and all other acquisition parameter information was extracted from the DICOM header files. To acquire segmentations, images were displayed with the lung viewing setting. Following physician annotations, each region of interest corresponding to a PN in the CT images was segmented using semi-automated contouring tools in Varian’s Velocity AI software by one of three raters.

Pixel data and segmentations were extracted from the DICOM files. Because radiomic texture features require voxels to be rotationally invariant [7] the PyRadiomics feature extractor [8] was used to resample images and masks to cubic-mm voxels. Lanczos interpolation was used for the images for greatest reproducibility [35] and nearest-neighbor interpolation was used for segmentation masks. To ensure all air and bone voxels were excluded and to improve reproducibility [36], voxels with intensities outside – 700 to 500 Hounsfield Units were excluded from calculations of non-shape features. Other radiomic studies of PNs have used narrower thresholds to analyze only malignant tissue [36] but we chose a less conservative threshold because we also analyzed benign tissue and very small PNs. All other PyRadiomics feature extractor settings were left at default values. A total of 107 features were extracted from all regions of interest.

### Formation of training and test sets for cross-validation of early detection models

In our baseline early detection pipeline, the early-development dataset was split into five folds using a randomized Group Stratified K-fold (scikit-learn) to ensure no patients overlap between training and test sets, repeated 10 times for a total of 50 unique training and test sets.

In our early detection pipeline with training set augmentation, we began with the same splits of the early-development dataset as used to validate the baseline model (50 unique training and test sets), and augmentation data were added to these training sets. For a given trial, scans were not used to augment the training set if the same patient appeared in that trial’s test set; such augmentation scans were unavailable to that trial. For each early-development train-test split, we varied the fraction of available augmentation data included in the corresponding training set: 5%, 10%, 15%, 20%, 25%, 50%, 75%, and 100% (8 unique fractions). Training set augmentation was cumulative. This gave a total of 50×8 = 400 unique augmented training sets corresponding to the 50 test sets.

### Harmonization of training and test sets

This study used OPNCB to correct for acquisition parameters. In OPNCB, harmonization is performed for each acquisition parameter iteratively in an order which removes acquisition dependency from the most features, as compared to other permutations [27]. Because ComBat is a data-driven method, no phantom imaging is required for this standardization [17].

We studied three methods of applying OPNCB to a given training set. In collective harmonization, OPNCB was applied to the complete training set, where extracted radiomic features and corresponding scan acquisition information were the inputs. In separate harmonization, the training set was separated by dataset before applying OPNCB to these subsets individually. In covariate harmonization, OPNCB was applied to the complete training set, and a dataset label for each scan was supplied as an additional input.

OPNCB was performed on training sets and the trained ComBat estimators were applied to the corresponding test sets using the function neuroComBatFromTraining being developed by Fortin et al. [14],[15]. The estimators appropriate to the acquisition parameters of a given test sample were applied in the order determined for the training set by the OPNCB algorithm. For separate harmonization, estimators applied to the test set were those learned for the early-development training scans alone.

ComBat fails if fewer than three training samples were acquired with the same protocol, e.g., if only two scans were acquired using a certain focal spot size. In such cases, these scans were excluded from the trial. If a protocol was used to acquire a scan in a test set but was not used to acquire any scan in the training set, that scan was also excluded from the trial, because no estimator was trained for that protocol. We developed an automated process to perform scan exclusion based on these criteria.

In our baseline early detection pipeline, OPNCB was applied using collective harmonization. In our early detection pipeline with training set augmentation, for each of our augmented training sets, we applied OPNCB using our three examined methods in parallel, leading to 3 versions of a given augmented training set and corresponding test set.

### Evaluation of acquisition dependence of radiomic features

In our early detection pipeline with training set augmentation, to evaluate whether harmonization removed acquisition dependence from augmented training sets, we used the Kruskal-Wallis test to compare feature distributions grouped by instances of acquisition parameters [17]. For example, feature distributions from CE scans were compared to those from non-CE scans. The test was run separately on the benign and malignant augmentation training data, because correct feature distributions should be consistent across acquisition within benign PNs or within malignant PNs, though benign and malignant distributions may differ. The test was not performed on the early-development training data because of this additional source of variability. For a given radiomic feature, if the test was significant (p ≤ 0.05) for either benign or malignant samples, that feature was deemed acquisition dependent. This procedure was applied to all 107 features.

To validate this procedure, we applied it to randomized splits of unharmonized scans that were acquired with the same protocols of interest—i.e. “acquisition-independent.” Our datasets contained 11 such uniform groups, ranging in size from 8 to 114 scans. These uniform groups contained both benign and malignant samples. The procedure defined an average 98.2% [97.8–98.5%] (n = 55) of features to be acquisition-independent as expected. This supports Kruskal-Wallis as a reasonable test to define acquisition dependence. Variability in unaccounted-for acquisition parameters most likely caused nonzero deviation from normal distributions.

### Feature selection and classifier training

To increase the generalization of a predictive model [37] and thus better model clinical applicability, we used a LASSO to select predictive features (regularization parameter α = 0.05) from each training set. Radiomic features were standardized to have zero mean and unit variance using the StandardScaler function from the Python library scikit-learn before LASSO was applied to select features. In our baseline pipeline, LASSO was applied to all radiomic features. In our pipeline with training set augmentation, LASSO was applied only to radiomic features which were acquisition-independent according to the Kruskal-Wallis test.

LASSO-selected standardized features from the training set were used to train a binary SVM (regularization parameter C = 1) [28] whose target was PN malignancy (class 0: endpoint benign diagnosis, class 1: endpoint malignant diagnosis). A linear kernel was chosen for the SVM to avoid overfitting. If harmonization failed or no features were selected in a given trial, an SVM was not trained for that trial.

Feature selection results and the trained SVM were applied to the held-out harmonized test set corresponding to each training set.

### Statistical analyses

We used Wilcoxon signed-rank tests to compare performance metrics between methods in trials where all models were trained. The Kruskal-Wallis test was adopted to compare feature distributions. Confidence intervals for continuous variables were calculated using the *t*-distribution and confidence intervals for discrete variables (e.g. numbers of selected features) were calculated using the Poisson distribution. These statistical tests were all conducted in Python using the SciPy library. The predictive performance of ML models was evaluated using the area under the receiver operating curve (ROC-AUC), weighted accuracy, sensitivity, and specificity, all of which were calculated using the Python library scikit-learn. The Delong test was used to compare ROC-AUC curves and was conducted in Python using the MLstatkit library.

All *p-*values reported in this study are two-tailed. Adjusted *p*-values were calculated with Holm-Bonferroni using the multipletests function from the Python statsmodels library to control for multiple comparisons across the three harmonization methods.

## Supplementary Material

Supplementary Files

This is a list of supplementary files associated with this preprint. Click to download.

• SciRepsupplement73125.pdf

## Figures and Tables

**Table 1:** Contents of the datasets. Patient and PN counts do not add to the column totals because augmentation datasets include follow-up scans of PNs in the early-development dataset.

	Patients		Pulmonary nodules		Scans	
	Count	% Cancer	Count	% Cancer	Count	% Cancer
Early-development	58	25.9	82	19.5	187	16.6
Augmentation benign	21	0.0	28	0.0	36	0.0
Augmentation malignant	58	100.0	59	100.0	189	100.0
Total	119	50.4	148	41.2	412	53.4

**Table 2:** Frequency of feature dependence on acquisition parameters. Frequency percentage measures the rate of acquisition dependence, defined as a significant Kruskal-Wallis test result, per acquisition parameter across all features and trials. Percentages do not sum to the given totals due to redundant failure. Sample numbers *n* are the 49 considered trials multiplied by 107 features.

	Unharmonized (%) (49 trials, n = 5243)		Collective harmonization (%) (49 trials, n = 5243)		Covariate harmonization (%) (49 trials, n = 5243)		Separate harmonization (%) (49 trials, n = 5243)	
	Benign	Malignant	Benign	Malignant	Benign	Malignant	Benign	Malignant
CE	27.1	79.4	58.7	89.1	23.4	30.6	0.0	9.3
Focal spot size	47.7	51.4	37.4	17.2	46.4	49.7	0.0	8.4
KVP	4.7	16.8	0.4	1.8	4.4	7.3	0.0	0.9
Manufacturer	0.0	47.7	7.3	10.2	26.7	23.7	0.0	0.9
Dataset total	53.3	84.1	80.1	90.7	65.3	68.8	0.0	13.1
Method total	87.9		97.4		87.6		13.1	

**Table 3:** Performance metrics for LASSO-SVM early detection models. Weighted accuracy uses balanced weighting by class (endpoint benign or malignant diagnosis).

	Collective harmonization (n = 45 trials)		Covariate harmonization (n = 49 trials)		Separate harmonization (n = 49 trials)	
	Mean, %	95% CI, %	Mean, %	95% CI, %	Mean, %	95% CI, %
Weighted accuracy	49.6	[48.7, 50.4]	67.2	[63.6, 70.8]	61.2	[56.9, 65.5]
Sensitivity	98.8	[96.3, 101.2]	52.9	[45.1, 60.8]	38.3	[30.5, 46.1]
Specificity	0.4	[−0.4, 1.1]	81.4	[78.1, 84.8]	84.0	[81.3, 86.7]

## Data Availability

The imaging datasets used in this study are currently restricted from public release due to data privacy laws and institutional review board policy. Anonymized radiomic feature data, acquisition parameters, and diagnostic data which were analyzed in this study are available from the corresponding author on reasonable request.
